# Supplementary material for: Are carbon emissions trading and green financial instruments synergistic? -Comprehensive quantitative research based on content analysis
Source: PLoS One. 2024 Mar 7;19(3):e0298601. doi: 10.1371/journal.pone.0298601 (PMC10919601; doi:10.1371/journal.pone.0298601)
Supplement: S1 Data — (ZIP) [file pone.0298601.s001.zip › Data/地级市Z_F工作报告.zip!/╕╩╦α/┬ñ─╧╩╨/└┤╫╘╣1⁄2╓┌║┼í╛┬φ┐╦╩2╛▌═°í┐_2018.docx]

各位代表：

现在，我代表市人民政府，向大会作工作报告，请予以审议，并请各位政协委员和列席人员提出宝贵意见。

市四届人大二次会议，是在党的十九大胜利闭幕、中国特色社会主义进入新时代的关键时期召开的一次重要会议。新时代开启了新征程，新时代凝聚着新力量。我们必须以习近平新时代中国特色社会主义思想为指导，不忘初心、牢记使命，始终把人民对美好生活的向往作为奋斗目标，始终把加快发展作为第一要务，锐意进取、苦干实干，以永不懈怠的精神状态和一往无前的奋斗姿态，为陇南人民谋福祉，为建设幸福美丽新陇南创造新业绩，继续向实现整体脱贫、实现与全省全国一道全面建成小康社会的目标奋勇前进。

2017年工作回顾

2017年，市政府全面贯彻党的十八大及十八届三中、四中、五中、六中、七中全会和十九大精神，深入学习贯彻习近平新时代中国特色社会主义思想,认真落实习近平总书记视察甘肃时的重要讲话和“八个着力”重要指示精神[1]，在省委、省政府和市委的坚强领导下，在市人大、市政协的监督支持下，团结带领全市人民，深化实施“433”发展战略，积极应对经济下行压力持续加大和自然灾害多发的严峻形势，统筹推进稳增长、促改革、调结构、惠民生、防风险等各项工作，全市经济运行总体平稳、社会事业全面进步、社会大局和谐稳定。

——经济运行趋稳向好。在全省经济逆势下滑的严峻形势下，预计全市生产总值达到359.1亿元、增长2.8%[2]。经济结构更趋优化，服务业增加值占生产总值的57.4%，消费对经济增长的拉动作用更加强劲。

——交通基础设施实现新突破。兰渝铁路全线通车，陇南成县机场建成试航，陇南人民的火车梦和飞天梦已经变为现实。

——生态环境更加良好。市区空气质量指数、全市水环境指数居全省第一，九县区城区水源地和主要河流水质达标率100%，森林覆盖率达到41.87%，高出了全省30.54个百分点。

——电商扶贫效应持续显现。陇南电商扶贫走在了全国前列，被确定为第三批国家电子商务示范城市，列入了中央政治局第39次集体学习成功案例之一。

——抢险救灾和灾后重建取得阶段性胜利。科学有效应对“8.7”暴洪泥石流、“8.8”九寨沟7.0级地震和“8.20”洪涝灾害，最大程度减少了灾害损失，监测预警、群众转移安置和抢险救灾、灾后重建等工作，得到了国家有关部委和省委、省政府的充分肯定，抢险救灾取得了重大胜利。

——人民生活不断改善。城乡居民人均可支配收入分别增长8%和10%，新增城镇就业2.4万人，城镇登记失业率、居民消费价格指数低于控制目标，人民群众生活水平有了新的提高。

我们主要做了以下工作：

一年来，我们深化供给侧结构性改革，经济发展质量效益同步提升。推进现代农业加快发展。粮食再获丰收，总产量达到110万吨。建成特色产业示范点100个、中药材标准化示范点46个，新增特色产业32.8万亩。建成规模养殖场32个，规模化养殖比重达到43%。新增省级龙头企业6家、农民专业合作社4651家[3]。礼县创建为国家级生态原产地产品保护示范区，祥宇橄榄油荣获纽约国际橄榄油大赛金奖，陇南茶叶荣获首届中国国际茶叶博览会设计银奖。工业经济企稳向好。认真落实一企一策方案，年初停产的29户企业已复产23户。实施市列重点工业项目20个，5个建成投产。创建省级中小企业公共服务示范平台3个，新增小微企业413户。非公经济增加值增长6.5%。编制了陇南经济开发区规划，实施基础设施项目24个，完成投资6.8亿元。第三产业快速发展。陇南特色农产品交易市场和12个便民市场建成运营。全域旅游、花满陇南创建活动成效显著，两当县名列“中国百佳深呼吸小城榜”第八位，宕昌县入围“2017最美中国榜”，康县花桥被评为第三批“中国乡村旅游创客示范基地”；官鹅沟5A级景区创建工作进展顺利，哈达铺、天池、金徽矿业创建为4A级景区；开通了兰渝铁路陇上江南号专列，旅游人数、综合收入大幅增长[4]。电子商务进农村综合示范县建设快速推进，网店达到1.3万家，华昌电商产业园、顺通电商物流园创建为省级生产性服务业示范企业、示范区。

一年来，我们聚焦特困片区精准发力，脱贫攻坚扎实推进。按照精细精确精微的“绣花”式扶贫要求，统筹推进片区扶贫、产业扶贫、电商扶贫、金融扶贫、旅游扶贫和社会扶贫，预计全年减少贫困人口10万人，贫困发生率下降到13.5%，两当县将实现整县脱贫退出验收。整合项目资金，推进国开行贫困县区农村基础设施建设项目，完成了农村公路“三年大会战”任务[5]，基本实现了建制村通畅工程全覆盖，西和县被命名为全国“四好农村公路”示范县；解决了33.9万人饮水不安全问题，实施农村D级危房改造2.5万户。完成易地扶贫搬迁4209户共1.7万人。大力推进产业扶贫，农业特色产业人均增收3100元，礼县荣获2017中国果业扶贫突出贡献奖。劳务输转68.3万人，创收141亿元。加快实施乡村旅游富民工程，创建国家级、省级乡村旅游示范村7个。放大金融扶贫效应，发放涉农贷款424.2亿元。创建电商扶贫示范网店2021家，带动贫困人口18万人。深入推进东西扶贫协作，落实帮扶资金9600万元。脱贫攻坚取得了重要阶段性成效。

一年来，我们强力推进项目建设，基础设施条件持续改善。坚持把项目建设作为夯基础、稳增长的重要举措，全面落实项目清单、并联审批、市级领导包抓项目责任制，实施投资规模500万元以上项目1192个。市财政筹资1500万元，加快了48个重点项目前期工作。积极做好征地拆迁和协调服务，渭武、徽两高速公路建设进展顺利，武九高速公路即将全线开工；13条普通省道、7条旅游公路开工建设；天平铁路南延线、市区轨道交通等项目前期工作顺利推进，“铁公机”综合交通网络基本形成。188个项目申报纳入国家PPP项目库。33个水利项目加快实施，成县磨坝峡水库、武都城区供水扩建等项目建成运营。投资14.5亿元的电信普遍服务试点项目加快实施，行政村有线宽带实现全覆盖。建成陇南大数据政务服务和乡村大数据系统。签约招商引资项目229个、总投资391亿元，到位资金180亿元。项目建设为加快发展创造了新的条件。

一年来，我们坚持建管并重，城乡建设步伐明显加快。启动了新一轮城市总体规划修编，康县、礼县县城及哈达铺、小川镇“多规合一”和195个乡镇规划编制全面完成，行政村村庄规划编制率达到85%。围绕打造“陇上江南”和“橄榄之城”，实施市区城市修补、生态修复工程，旧城区风貌改造和绿化、美化、亮化等工作初见成效。加快推进东江新区外环路、十号路、西和城北棚户区改造和各县城区公园广场、道路桥梁、停车场等项目，东江新区福津北路、两水后坝水源地、武都城区滨江步道改造等项目建成投用，开展污水处理厂运营达标和生活垃圾无害化处理设施建设突破行动，市政基础设施更趋完善。实施棚户区改造2422套、重点村镇风貌改造2.3万户。房地产项目完成投资17亿元。完成了土地利用总体规划修编、永久性基本农田划定工作。全面实施全域无垃圾创建活动[6]。建成生态文明新农村418个。全市城镇化率预计达到32.8%，城市化水平有了新的提高。

一年来，我们着力加强生态环境保护，生态文明建设快速推进。围绕构建长江上游生态安全屏障，大力推进生态文明示范市建设，加快实施水生态文明城市建设试点、“两江一水”综合治理等项目。全面推行了河长制，编制完成11条河流“一河一策”方案和任务清单。严格落实环境保护“党政同责、一岗双责”责任制，成立了长江上游生态屏障安全和保护巡察办公室。积极整改中央环保督察组和省环保督察组反馈问题，开展了自然保护区违法违规项目、重点排污企业污染防治、河道污水垃圾及采石采砂清理专项整治活动，水、大气、土壤污染治理和环境保护进一步加强。落实安全生产责任制，按照“一库一策”方案，完成了69座病险尾矿库综合治理，生态环境有了明显改善。

一年来，我们持续深化改革开放，发展活力不断增强。深入推进“接放管服”改革，行政审批事项减幅58.1%。市场主体达到12.5万户，增长6.7 %。积极推进财税体制改革，减免退税9.7亿元。创新“3+1”金融服务体系，市县乡村四级产权交易平台联网运营，金融支撑保障更加有力[7]。启动了农村“三变”改革[8]，农村土地承包经营权确权登记通过省级验收。教育、卫生体制和户籍制度改革、供销社综合改革进展顺利。加强对外交流合作，与白俄罗斯格罗德诺市缔结为友好城市，成功举办了第26届西交会、第9届乞巧女儿节、第3届白马人民俗文化旅游节，陇南的知名度和影响力得到了不断提升。

一年来，我们积极防范和科学应对，抢险救灾和灾后重建取得阶段性成效。在党中央、国务院的亲切关怀和省委、省政府的有力指导下，市委、市政府快速反应、科学应对，迅速组织各级党政组织和广大干部群众齐心协力抢险救灾，最大限度地减少了灾害损失。坚持把群众安置放在第一位，及时下拨救助资金7050万元，解决临时居住和越冬等生活困难问题，确保了受灾群众有饭吃、有衣穿、有干净水喝、有安全住所、有病能医。迅速启动灾后重建，编制完成了灾后恢复重建规划，已经省政府常务会议审定实施。积极汇报争取重建政策和项目资金，5596户群众住房维修任务基本完成，4923户群众住房重建已经开工，基础设施和公共服务设施、地质灾害治理等灾后重建项目正在有力有序推进。

一年来，我们坚持抓保障惠民生，社会事业协调发展。始终保持为民情怀，市、县财政支出的80%用于保障和改善民生，一大批惠民举措有效落实，人民群众的获得感、幸福感明显增强。加快县域义务教育均衡发展，新改建村级幼儿园161所，高考二本以上上线率达到28.1%。推进公共卫生服务均等化，新农合参合率达到98.4%，创建医养结合示范点19个。加强公共文化服务体系建设，村级文化综合服务中心实现全覆盖。社会保障体系日益健全，机关事业单位养老保险、特困人员供养、医疗救助等政策全面落实，城乡低保标准逐年提高。建成“双创”园区11个、创业孵化基地9个。10件省列、8件市列民生实事基本办结。坚持加强和创新社会治理，深入推进“双查双整”集中行动和平安陇南建设，全市社会大局和谐稳定。民族宗教、外事侨务、统计、审计、档案、地方志、气象、地震、“双拥”、人防、粮食安全、质量监管、人才管理和妇女儿童、残疾人、老龄、慈善、红十字会、关心下一代等工作都取得了新的成绩。

一年来，我们持续加强自身建设，政府效能不断提升。坚持依法行政，全面落实省委、省政府和市委决策部署，自觉接受市人大及其常委会的法律监督、工作监督和市政协的民主监督，105件人大代表建议、231件政协委员提案全部办结。坚持科学民主决策，修订了《陇南市人民政府工作规则》，成立了市政府决策咨询委员会、重大行政决策廉政监督委员会[9]。“两学一做”学习教育常态化制度化扎实推进。加强行政监察和审计监督，深入开展了“三纠三促”专项行动[10]。全面落实党风廉政建设主体责任，严格执行中央“八项规定”、省委“双十条”和市委各项规定，政府系统党风廉政建设得到了明显加强。

各位代表，过去一年的成绩来之不易。这些成绩的取得，是省委、省政府坚强领导、亲切关怀的结果，是市委统揽全局、科学决策的结果，是市人大及其常委会、市政协及社会各界有效监督、倾力支持的结果，是全市广大干部群众和衷共济、奋力拼搏的结果。在这里，我代表市人民政府，向奋战在各条战线上的广大干部群众，向省驻陇南各单位、人民解放军和武警驻陇南部队官兵，向各民主党派、人民团体、工商联、无党派人士、离退休老同志，以及所有关心、支持陇南发展的社会各界人士表示崇高的敬意和诚挚的感谢！

各位代表，党的十九大作出了中国特色社会主义进入新时代、我国社会主要矛盾已经转化为人民日益增长的美好生活需要和不平衡不充分的发展之间的矛盾等重大政治论断。我们要奋进新时代、开启新征程。走进新时代，意味着陇南发展的基础条件已经发生了巨大变化。通过历届市委、市政府和全市人民的长期不懈努力，我市综合实力明显增强，城乡面貌发生了历史性巨变，交通等基础设施实现重大突破，人民群众的幸福感、获得感不断增强。电商扶贫创出了全国品牌，美丽乡村、文化旅游、金融创新、生态文明建设等工作有了突破性发展，为全省乃至全国创出了可复制、可借鉴、可推广的经验。这些，为我们进入新时代，建设幸福美丽新陇南奠定了坚实基础。我们对新时代发展充满信心。

建设幸福美丽新陇南，是历史赋予我们的神圣责任和使命。奋进新时代，意味着陇南加快发展的任务更加艰巨而繁重。我市发展不足、欠发达的基本市情还没有根本改变，发展不平衡不充分的问题还很突出，主要表现在：经济总量小，县域经济综合实力不强，调结构、促转型的压力很大；贫困面大、贫困程度深，城乡居民收入偏低，脱贫攻坚的任务十分艰巨；招商引资引进的大项目、产业链项目少，重大项目谋划不足，前期工作滞后，年初确定的一些重大项目、重点工作任务没有达到进度要求，经济逆势下滑的态势还没有从根本上得到扭转；自然灾害易发频发，防灾减灾、安全生产和环境风险管控压力依然很大；公共服务保障能力不强，城乡基础设施建设还有欠账，群众行路难、过河难和城市脏、乱、堵等问题还没有彻底解决；部分单位和工作人员担当意识不强、办事效率不高、抓落实不力的问题仍然存在。这些，都是我们进入新时代必须破解的难题和工作的着力点。我们要以舍我其谁的责任担当、奋发有为的精神状态，撸起袖子加油干，锲而不舍抓落实，更好地满足人民群众日益增长的美好生活需要，让陇南人民真正幸福起来。

新时代要有新气象，新时代更要开创新业绩。当前，陇南的发展站在了新的历史起点上，加快发展、坚决打赢脱贫攻坚战、建设幸福美丽新陇南是全市人民共同的心声和期盼，每一个陇南儿女都有责任和义务，在这场伟大的实践中贡献自己的智慧和力量；每一个陇南儿女，都要全力支持改革，全力支持发展。我们坚信，只要全市各级组织和广大人民群众不忘初心，奋力拼搏，就一定能够如期实现整体脱贫，与全省全国一道全面建成小康社会，就一定能够在建设幸福美丽新陇南中，一起奋进接力，谱写出新的壮丽篇章！

2018年主要目标及工作任务

根据党的十九大关于新时代中国特色社会主义发展的“两个阶段”战略安排[11]，结合陇南实际，总的考虑是，到2020年，现行标准下全市农村贫困人口实现脱贫，九县区全部摘帽，与全省、全国一道全面建成小康社会；再奋斗15年，到2035年，全市县域经济实力大幅跃升，基础设施更加完善，生态环境更加良好，城乡居民收入、基本公共服务达到全省平均水平，与全省全国一道基本实现社会主义现代化；再奋斗15年，到本世纪中叶，物质文明、政治文明、精神文明、社会文明、生态文明全面提升，全市人民共同富裕的目标基本实现，把陇南建成富强民主文明和谐美丽的现代化城市。

2018年是深入学习贯彻党的十九大精神的开局之年，也是决胜全面建成小康社会、实施“十三五”规划承上启下的关键一年，做好今年的政府工作，意义特殊、责任重大。政府工作的总体要求是：全面贯彻党的十九大和十九届一中全会精神，深入学习贯彻习近平新时代中国特色社会主义思想和视察甘肃时“八个着力”重要指示精神，坚持稳中求进工作总基调，牢固树立和贯彻新发展理念，按照高质量发展要求，统筹推进“五位一体”总体布局和协调推进“四个全面”战略布局，全面落实省委省政府决策部署和市第四次党代会精神，坚持有所为有所不为，扬长避短、后发赶超，深化实施“433”发展战略，加快推进经济、政治、文化、社会、生态文明和政府自身建设，以供给侧结构性改革为主线，以项目建设为抓手，突出抓重点、补短板、强弱项，坚决打赢精准脱贫、防范化解重大风险、污染防治、棚户区和农村危房改造、生态文明新农村建设、灾后重建攻坚战，大力发展县域经济，加快建设幸福美丽新陇南。

2018年全市经济社会发展的主要预期目标是：生产总值增长5.2%，固定资产投资增长5%，规上工业增加值增长5.8%，社会消费品零售总额增长9%，一般公共预算收入增长6%，城镇居民人均可支配收入增长8%，农村居民人均可支配收入增长11%，居民消费价格指数、单位生产总值能耗、污染减排控制在省定目标以内。围绕实现上述目标，重点抓好11个方面的工作：

一、坚决打赢脱贫攻坚战。坚持精准扶贫精准脱贫，做到脱真贫、真脱贫，是我们肩负的一项重大政治责任。我们要以精准扶贫精准脱贫统揽全局，紧盯“两不愁、三保障”，瞄准1365个建档立卡贫困村和剩余贫困人口，下足精细精确精微“绣花”功夫，快速推进脱贫攻坚，确保两当县稳定脱贫，成县、徽县达到整县退出验收标准，全市稳定脱贫10万人以上。

把深度贫困村打造成美丽新农村。脱贫攻坚是一场硬仗，打赢深度贫困村脱贫攻坚战更是硬仗中的硬仗，必须下大力气、坚决攻克深度贫困村这个堡垒。今年，要整合90%以上的财政扶贫资金和80%以上的涉农项目资金，市级财政收入增量的10%、县区20%增列为扶贫专项资金，统筹用于1154个深度贫困村脱贫攻坚。积极争取和用好农村基础设施和人居环境综合治理专项贷款[12]，加快实施贫困村整体提升工程、农村人居环境综合治理项目，全面推进村组道路建设，解决7.1万人饮水不安全问题，实现标准化村卫生室和幼儿园全覆盖，整体改善农村发展条件。坚持政府引导、群众为主、统一规划、分类指导，推广康县模式，全力解决农村基础设施、公共服务设施短板问题，力争把最贫困的561个村建成美丽新农村[13]，再用两年时间，彻底消除深度贫困村。

切实解决好深度贫困群众增收难题。坚持分类指导、因户施策，用足用好产业扶贫专项贷款、产业发展投资基金[14]，实施“六个一批”工程，促进贫困群众稳定增收。产业发展脱贫一批。坚持市场导向，培育壮大农业特色产业，健全农产品加工、仓储、营销体系，扶持引导川坝河谷区、特色产业主产区贫困群众率先脱贫奔小康。土地流转脱贫一批。围绕特困片区建设60个农业特色产业园（区）[15]，鼓励引导龙头企业、专合组织和能人大户，以土地流转托底为重点，大力发展适度规模经营，带动贫困群众通过流转土地、企业用工实现稳定增收。公益岗位脱贫一批。在深度贫困村长期设立乡村道路养护员、环境卫生保洁员、自然灾害预警员、公益设施管理和治安联防员、社会保障协管和孤寡老人留守儿童看护员等公益性岗位5万名，实现“三无”特困家庭增收脱贫[16]，统筹解决全域无垃圾创建、乡村公路管护、农村矛盾纠纷化解和治安管理、农村孤寡老人和留守儿童管护、自然灾害预警等方面的问题。生态补偿脱贫一批。争取落实生态护林员1万名，在易地扶贫搬迁原址、高山区域和荒山荒坡进行植树造林和生态林管护，解决深山林缘地区贫困群众增收问题。劳务输出脱贫一批。加强劳动技能培训和劳务对接，促进劳力型、分散型向技能型、有组织输出转变，实现长期务工增收脱贫。政策兜底脱贫一批。坚持规范认定、公开透明、动态管理，不断提高农村低保救助标准，对特困家庭实现“政策性兜底脱贫”，确保整体脱贫、全面小康路上不漏一户、不落一人。

加快推进农村危房改造和易地扶贫搬迁。把解决农村群众不安全住房作为精准扶贫的重中之重，完成省上下达的农村危房改造任务，基本消除农村土坯危房，实现贫困户住房安全。创新搬迁模式，集中安置搬迁一批、村内就近搬迁和插花搬迁一批、小城镇和中心村集中安置一批、货币化安置一批、省外搬迁一批，完成易地扶贫搬迁0.8万户3.4万人。统筹推进安置区水、电、路等基础设施和教育、文化、卫生等公共服务设施建设，因地制宜发展壮大富民产业，确保搬迁群众搬得出、稳得住、能发展、可致富。

深入推进东西扶贫协作。以深度贫困村为重点，推进青岛对口区市街镇、社区、企业联乡包村，实施一批基础设施和特色产业开发、劳务协作项目，打造东西扶贫协作精准帮扶示范区、示范村。加快建设青岛陇南产业园，引进一批青岛企业落户园区，把青岛陇南产业园打造成东西扶贫协作示范基地。利用青岛沿海城市优势，借船出海、贴牌上市，让陇南的农特产品走出深山、走向全国。加强与青岛用工市场对接，有组织输转农村劳动力到青岛就业。进一步深化教育、医疗、人才培训等方面的协作，促进东西扶贫协作效益最大化。

新时代赋予我们脱贫攻坚的神圣使命。我们必须举全市之力，坚决打赢精准扶贫精准脱贫攻坚战，我们也一定能够打赢这场艰巨的脱贫攻坚战。

二、着力打造秀美宜居的魅力城市。城镇化是现代化的必由之路，是推动区域协调发展的有力支撑。我们要围绕构建以县城为中心、中心乡镇和特色小镇协调发展的现代化城市，快速推进城乡一体化，把陇南打造成绿色、美丽、和谐、宜居、宜游、宜商的美丽家园。

优化城镇布局和形态。突出美丽陇南、陇上江南城市定位，统筹人口、产业、交通、生态等要素，加快修编城市总体规划，在2020年实现全面小康的基础上，到2035年把陇南建成连接“一带一路”和长江经济带的重要节点城市，打造成融合陕甘川元素的特色城市、长江上游重要的生态屏障，使美丽陇南真正成为活力之城、生态之城、陇蜀之城。按照“东西扩展、连接南北、城市双修、整体提升”的思路，加快推进武都市区“双修工程”，抓好汉王、吉石坝、两水新区开发，启动安化、马街和姚寨特色小镇建设，把武都打造成中国橄榄之城，建成全市的政治中心、文化旅游中心、金融中心和商贸物流中心。依托陇南经济开发区，建设徽县成县快速通道，推进产城融合发展，把徽县、成县建成全市的经济中心。强化县城带动功能，逐步推进西和、礼县一体化，扩大文县、宕昌县、康县、两当县的城市规模，加快新区建设，推动产业分工、基础设施、生态保护、环境治理协调联动，培育形成更多支撑区域协调发展的增长极。加快中心乡镇和特色小镇建设，以县城周边中心镇、旅游乡镇为重点，打造一批充满魅力、彰显特色、生态优美的精品小镇。

加强城市基础设施建设。坚持适度超前、综合配套、和谐宜居，统筹推进市区和各县城区基础设施建设。加快武都旧城区改造和新区开发，启动莲湖、县门街片区拆迁改造和滨江体育场建设，实施长江大道东延伸段及地下管廊建设，加快推进东江新区外环路、十号路及站前广场安置区、东江新区供水和污水处理厂异地扩建、供热二期、天然气门站等市政基础设施建设，进一步提升市区综合服务功能和城市品位。坚持强基础、补短板、提品质、显特色，大力实施各县城区和重点镇道路桥梁、地下管网、停车场、公园绿地和集中供热、供水、污水垃圾处理等项目。加快智慧城市建设，健全文化体育、旅游、社区生活服务设施，让群众生活得更安心、更省心、更舒心。

保障房地产市场健康发展。坚持房子是用来住的、不是用来炒的定位，健全房地产市场平稳健康发展的长效机制，使房价回归在合理区间。把棚户区改造与旧城改造、新区开发和小城镇建设结合起来，实施棚户区改造4231套，建设一批中高档住宅，启动实施保障性住房和乡镇干部周转房、中心乡镇创业门店等项目，更好地满足群众住房需求。加快建立多主体供应、多渠道保障、租购并举的住房制度，对无力购买住房的居民、非户籍人口，支持其租房居住，对特困家庭给予租金补助，做到居者有其屋、住房有改善。全面推广街区制[17]，开放封闭住宅小区，对已建成的封闭式机关单位逐步打开院墙，实现内部道路公共化，形成开放便捷、配套完善、绿色共享、邻里和谐的生活街区。

加快农业人口向城市转移。坚持以人为本、城乡统筹，加快推进户籍制度改革，让有能力在城镇稳定就业和生活的农业人口举家进城落户，与城镇居民享受同等权利和义务。优先解决农村大中专毕业生、农村参军及退伍军人、已在城镇就业居住的农业人口和农民工进城落户问题。鼓励农民带着土地进城，切实维护好落户农民的土地承包权、宅基地使用权、集体收益分配权，支持引导落户群众依法自愿有偿转让。全面推行居住证制度[18]，保障居住证持有人享有文化教育、医疗卫生、就业养老等基本公共服务，实现未落户常住人口居住证全覆盖。全市城镇化率达到35%以上。

三、加快工业经济转型升级。经济开发区是推动工业化、城镇化快速发展和对外开放的重要平台。我们要以陇南经济开发区为引领，示范带动各县区经济开发区建设，引导工业项目向开发区集中，促进产业集聚、资源集约、绿色发展，快速推进工业经济提质增效和转型升级。

全力推进开发区建设。坚持徽成一体化、产城融合发展，高标准编制陇南经济开发区规划，全面推进道路桥梁、污水垃圾处理、供水、供热、供气、通讯、金融服务等基础设施建设，启动实施临港经济区、保税物流中心、商业综合体、工商学院和陇南师专分校、职业技术学院、金徽酒城等项目，把陇南经济开发区培育成项目集聚、资源集合、产业集群的重要战略平台。围绕重点发展方向和特色优势产业，引进5户以上大企业、大集团落户陇南经济开发区，培育形成支撑全市经济发展新的增长极。加强与汉能集团的务实合作，开工建设陇南汉能移动能源产业园。加快发展“飞地经济”[19]，每个县区谋划2个投资亿元以上的工业项目落地陇南经济开发区，带动县域经济发展。加快各县区经济开发区、工业园区建设，配套完善基础设施，促进产业聚集发展。

加快传统工业转型升级。深入实施工业强市战略，坚持提质增效与扩大总量并重，扶持引导现有铅锌、黄金和农产品加工等资源型工业企业加大技术改造，实现转型升级。大力发展有色金属新材料、装备制造和绿色农产品精深加工产业，做大做强白酒酿造产业，进一步提升工业发展质量。坚持绿色发展理念，大力发展清洁能源、现代制药、医养保健等战略新兴产业，打造绿色工业品牌。加强监测调度，扶持帮助停产企业恢复生产，力促骨干企业满负荷生产，确保工业经济企稳回升。强化企业创新主导作用，加快企业技术创新，扶持发展一批上市公司，力争培育4户年销售收入10亿元以上的骨干企业、25户年销售收入亿元以上的成长性企业，规上工业企业达到90户以上。

加快重点工业项目建设。积极培育新的工业增长点，实施重点工业项目28个、总投资64亿元[20]。力争恒丰核桃、华康杜仲、金鑫工贸新材料、金徽酒制曲技改等项目建成投产；促进郭家沟铅锌矿采选、青峰石材扩建等项目达产达标；加快厂坝公司300万吨技改扩能、祥宇油橄榄生态工业园、礼县鑫晟源大黄等续建项目进度，及早建成投产；抓好成州九源核桃、红川酒业扩能、宝徽锌冶炼废渣回收及环境治理、独一味工业园区扩建等项目前期工作，力争年内落地实施，增强经济发展新动能。

大力发展非公经济。构建“亲”“清”新型政商关系，改善营商环境，激发全社会的创业激情，激活各类资源要素，培育更多的市场主体，实现非公经济在数量、质量、结构、效益上取得新突破。新增中小微企业450户以上，省级“专精特新”中小企业达到5户，省级中小企业公共服务示范平台达到10户[21]，甘肃股权交易中心托管挂牌企业达到75户以上。

四、加快推进农业农村现代化。农业是稳民心、安天下的第一产业。必须始终把解决好“三农”问题作为重中之重，坚持农业农村优先发展，深化农业供给侧结构性改革，快速推进农业农村现代化。农业增加值增长5%以上。

实施乡村振兴战略。把统筹城乡发展作为重中之重，科学制定乡村振兴战略规划，健全城乡融合发展机制，发展各具特色的城乡产业体系，形成以产促城、产城融合、城乡一体的发展格局。按照产业兴旺、生态宜居、乡风文明、治理有效、生活富裕的总要求，新建生态文明新农村200个、省级美丽乡村示范村23个[22]。抓好17个特色小镇建设[23]。实施重点村镇风貌改造项目。深入开展全域无垃圾创建活动，完善城乡垃圾污水收集处理系统，集中整治城乡交通秩序、环境卫生、乱搭乱建、占道经营等问题，整体改善城乡人居环境。

加快发展多种形式的适度规模经营。坚持质量兴农、绿色兴农，在确保粮食安全的基础上，加快标准化特色产业基地建设，着力培育壮大核桃、花椒、油橄榄、中药材等特色主导产业，新增经济林果17万亩，完成综合管理650万亩，推广中药材标准化种植47万亩。加快发展规模高效养殖，发展生态放养鸡550万只、中蜂养殖30万箱，新建标准化养殖场（小区）30个。因地制宜发展苹果、茶叶、食用菌、苗木等区域优势产业，把地方土特产和小品种做成带动农民增收的大产业。组织好花椒、核桃、苹果、中药材、苗木等产销对接和电商洽谈活动，健全营销体系，把陇南的绿色“宝贝”销往全国，打造成群众脱贫致富的铁杆庄稼。

大力发展新型农业经营主体。组建陇南富民产业发展公司，通过土地流转、股份合作，带动龙头企业、家庭农场、能人大户等新型经营主体做大做强，与农户建立利益共享、风险共担的联结机制，逐步实现小农户和现代农业发展有机衔接。培育发展龙头企业和新型经营主体100个以上。大力发展农民专业合作社，在规范运营、基础投入、产业升级、品牌建设等方面大力扶持，新增市级以上示范社30个。引导合作社通过联合、重组、参股经营等方式，整合各类资源，推进农业特色产业标准化种植、集约化经营、产业化发展，促进农业增效、农民增收。

积极创建田园综合体。坚持政府引导、企业参与、市场化运作，以省级美丽乡村示范村为重点，开展试点示范，积极打造一二三产业联动、生产加工销售旅游一体、产村融合的田园综合体，示范带动现代农业发展。立足资源禀赋和市场需求，组建产业公司，整合项目资金,加快建设徽县泥阳现代农业、文县李子坝茶叶、礼县苹果矮砧密植等现代农业示范园[24]，在“两江一水”流域建设50万亩高效露地冬早春蔬菜生产示范区，创建特色农业示范点100个，构建特色鲜明、链条完整、布局合理、生态环保、多功能融合的现代农业产业体系。谋划实施一批农产品精深加工项目，形成“龙头带动、农民参与、促农增收”的利益联结机制，推进贸工农、产加销统筹衔接，实现农村一二三产业融合发展，着力打造现代休闲农业、观光农业、生态农业示范区。

五、加快发展南向通道经济、电子商务和全域旅游。加快向南开放步伐，主动融入“一带一路”，是陇南加快开放型经济发展的必然趋势。我们要抢抓兰渝铁路、陇南成县机场建成机遇，快速推进南向通道经济[25]、电子商务和全域旅游等现代服务业发展，真正把陇南建成甘肃向南开放的桥头堡。

构建向南开放新格局。积极抢抓中新(中国与新加坡)互联互通南向通道建设重大机遇，深化与毗邻市州交流合作，加快推进基础设施互联互通、产业培育协同协作、城乡发展共建共享、生态环境联防联治、经济发展优势互补，主动融入长江经济带、成渝经济区、关天经济区和大九寨经济圈，加快形成全方位向南开放开发新格局，把陇南打造成连接丝绸之路经济带和长江经济带的战略新通道、西北最便捷的向南出海大通道。

健全现代商贸物流体系。以服务消费、产业转型和城市发展为支撑，做大做强融资租赁、家政养老、信息服务等现代服务业，改造提升交通运输、批发零售、餐饮等传统服务业，增强消费对经济发展的基础作用。进一步完善武都吉石坝现代物流园、宕昌哈达铺现代物流园、陇南电商孵化园基础设施和配套服务功能，加快推进陇南空港物流园、物流集散中心建设，开工建设陇南会展中心、哈达铺华昌药材物流城二期、宕昌中药材现代仓储物流园、文县物流采购配送中心和城乡便民市场，把陇南建成南向通道经济带上的重要物流节点城市、连接西北西南的物流集散中心。

全力创建国家电子商务示范城市。以做大网商、做优品牌、做精网货、做强市场为重点，加快推进国家电子商务进农村示范县、阿里巴巴千县万村项目建设，进一步提升电商运营能力和服务水平。组建成立电商投融资公司，大力扶持精品网店，创建陇南特色网货供应平台，建立区域电商物流配送中心，进一步健全电商综合运营体系。以做大做强特色产业为支撑，大力发展标准化种植基地，培育壮大精深加工龙头企业，做到一户电商发展一个加工企业、一个生产基地，加快推进电子商务与实体经济、精准扶贫深度融合。在电子商务上集中突破，拓展“互联网+”、“物联网”在工业、旅游、文化、物流、公共资源交易、政府购买服务、城市共享经济等方面的应用与发展，让电商真正成为引领陇南发展的新潮流。

大力发展全域旅游。在旅游开发上集中突破，加快大景区建设，深入挖掘陇南文化元素，着力打造陇上江南旅游品牌。康县、宕昌县创建为国家级全域旅游示范县，成县、两当县创建为省级全域旅游示范县。加快官鹅沟5A级景区创建进程，建成官鹅沟自驾游房车营地、客运索道和康县茶马康城、花桥温泉宾馆、文县天池大景区等旅游项目，创建4A级景区1家。启动实施陇南旅游集散中心、阶州印象和大熊猫国家公园、文县白马人民俗风景区、阳坝文化自然遗产保护与开发、官鹅沟大景区快速通道、西狭颂文化养生小镇、大堡子山遗址公园、祁山武侯祠旅游开发、陇蜀古道、茶马古道遗址等文化旅游项目，完善景区宾馆饭店、娱乐购物、道路、停车场、公厕、标牌标识等配套设施，大力提升旅游景区服务品质，真正让景区活起来、火起来。加快发展观光、休闲、度假、医疗养生等多元化的乡村旅游产业体系，创建乡村旅游示范村125个[26]。积极融入大九寨旅游经济圈，开辟陇南旅游精品线路，做大做强生态养生、历史文化、乡村休闲、民俗风情等旅游品牌，发展以宕昌县为主的冰雪旅游，创建独具魅力、功能完善的旅游目的地。全年旅游接待人数、综合收入分别增长20%以上，把文化旅游业打造成陇南绿色发展崛起的主导产业。

六、全力抓好项目建设。项目是拉动投资、加快发展的主要抓手，是改善民生、造福百姓的支撑点。我们要牢牢抓住项目建设这个“牛鼻子”，围绕补短板、促转型，着力夯实硬件基础，谋划实施一批绿色环保、改善民生、利于长远的大项目、好项目，以项目拉动投资、培植财源、带动就业、促进发展。

谋划实施一批重大项目。全力打好项目攻坚战，今年谋划实施投资规模500万元以上项目1308个、总投资3627亿元，力争完成投资600亿元以上[27]。进一步完善综合交通运输体系，力争开工建设礼县至洛门、余家湾至文县、望关至阳坝等高速公路和徽县成县快速通道、武都区城市轨道交通、宕昌县毛雨山隧道等重大交通项目，武九高速全线开工建设，徽两高速建成通车，渭武高速实现区域性通车；抓好武都裕河至两河口等11条普通省道升级改造项目，加快建设宕昌至官鹅沟、成县小川至西狭等6条旅游公路[28]。争取兰渝铁路开通动车组，天水至陇南铁路开工建设，积极推进阳平关至陇南至九寨沟、合作至哈达铺至徽县、兰州至天水至汉中等铁路项目前期工作。加快建设武都区、礼县、宕昌县3个通用机场和4个直升机起降点，陇南成县机场开通青岛、重庆、上海、北京等航线。加快水利项目建设，抓好节水灌溉和江河干支流、中小河流、山洪沟道治理、城区和重点镇水源地保护、供水基础设施项目建设，开工建设徽县宋家湾水库，加快礼县永固峡水库前期工作，建设修复一批“五小水利”工程，切实提升水利保障能力。抓好能源通信基础设施项目建设，大力发展风电、光电、生物质等新能源，建成武都千坝风电一期、武都徽县330千伏变电工程。加快推进天然气管网、加气站和户用工程。积极开展5G网络建设试点。抓好大数据基础设施建设，建成陇南云中心，拓展应用领域，为创建智慧城市增添新活力。

着力破解项目建设难题。加快项目前期工作。对谋划项目抓紧开展可行性研究、初步设计、咨询评估、项目审批、招投标等前期工作，确保今年3月份前开工一批重大项目。加快项目审批。全面推行项目清单制，对完成土地预审和选址等审批要件的项目，开通“绿色通道”，实现项目在线审批“一次登录、全网通办、全流程监督”。破解项目建设融资难题。全面启动陇南经济开发区基础设施等PPP项目，对纳入国家PPP项目库的7个项目，抓紧开展前期论证、设计和包装推介，力争PPP项目建设实现新突破；用好地方政府债券，引导保险资金以股权、债券、不动产投资等方式参与项目建设，民间资本和社会投资增长5%以上。着力破解征地拆迁难题。切实维护好群众的合法权益，优先保证重大项目、民生工程建设用地，做到依法和谐征迁。

加大招商引资力度。积极组织参加兰洽会、西洽会、西博会、渝洽会、西交会等重大节会，开展产业链招商、一对一招商、点对点招商、以商招商，做到签约一个、开工建设一个。认真落实招商引资优惠政策，实行重点招商项目清单化管理，对近三届兰洽会、第26届西交会签约未落地项目逐一跟踪落实，扩大有效投资，推动实体经济发展。

七、持续加强环境保护和安全生产。人与自然是生命共同体，保护好自然环境就是保护人类自身安全。我们要牢固树立绿色发展理念，把环境保护、安全生产与健全监管执法体系结合起来，围绕构建长江上游重要的生态安全屏障，快速推进生态文明建设，为保障国家生态安全做出新的更大贡献。

加强生态系统修复和保护。积极开展国土绿化行动，加快实施“两江一水”区域综合治理、生态文明示范市、水生态文明城市试点项目，建设城市、社区绿色公园，规划建设和分步实施武都北山公园，进一步提升绿色服务功能。完成退耕还林还草12.8万亩、绿色通道350公里，抓好重点公益林、天保工程管护任务。全市森林覆盖率达到41.9%以上。开展生态红线、永久基本农田、城镇开发边界三条控制线划定工作。推行国土空间用途管制，提高节约集约用地水平。坚持谁保护谁受益、谁污染谁治理，健全生态补偿机制。严格落实河长制，统筹推进生态环境保护、河道综合治理、污水垃圾处理、沿河经济发展，切实做好“水”“岸”两篇文章，着力打造沿河生态旅游线和经济带。

打好污染防治攻坚战。实行最严格的环境保护制度，持续推进大气、水、土壤污染防治工作，确保空气环境质量进一步改善，主要河流水质稳定达标，城乡居民饮水安全。全力抓好中央环保督察反馈问题后续整改、省级环保督察反馈意见的整改工作。深入开展自然保护区、森林公园、湿地公园和大熊猫国家公园生态环境问题专项整治行动。完善铅、锌、锑等重金属和黄金类污染物应急处置预案，加强应急演练，提高应急处置和防范能力。积极倡导简约适度、绿色低碳的生活方式，深入开展节约型机关、绿色家庭、绿色学校、绿色社区和绿色出行创建活动，努力让全市人民共享更美好、更舒适、更优质的生活环境。

切实抓好安全生产工作。安全生产没有“保险箱”。我们要牢固树立生命至上、安全第一的思想，时刻绷紧安全生产这根弦，持续推进重点行业、重点领域安全隐患排查整改，从源头上预防安全事故发生。筹办好全国尾矿库综合治理现场会。加快推进安全生产信息化，实现尾矿库视频监控系统全覆盖，在农村道路、学校、人员密集场所和旅游景点、乡镇街道、建筑工地等重点领域安装在线监控系统。组织开展消防、防洪、防灾、防恐、安全生产等各类应急演练，企业负责人、安全管理员、企业员工培训率和特种作业人员持证上岗率分别达到100%，让安全意识深入人心。

健全环保安全执法监管体系。切实发挥好长江上游生态屏障安全和保护巡察办公室的职能，按照全覆盖、专业化、常态化监管要求，提高执法能力和装备技术水平，建立监管信息平台，形成责任到人、监管到位、执法规范、高效运转的监管格局，实现环境保护、安全生产监管执法全覆盖。把专项整治与经常性执法检查结合起来，及时排查整治重点行业领域存在的环境安全隐患及突出问题。严格落实“党政同责、一岗双责、失职追责”责任制，推行环境保护、安全生产清单制，做到尽职免责、失职问责。环境就是民生，青山就是资源，蓝天也是财富。我们要切实保护好生态环境，让陇南的水更清、山更绿、天更蓝。

八、切实强化财税金融支撑保障。财政金融是经济发展的“晴雨表”。我们要始终把财税金融工作作为促发展、保运转、惠民生的首要任务，切实抓好开源、节流、服务、监管工作。

加强财源建设和税收征管。用好财源建设基金，加大对新兴产业、中小微企业的扶持力度，培育壮大规上企业，大力发展现代服务业、商贸物流业，形成新的税源增长点。加强重点建设项目及有色金属采选冶、建筑、房地产、白酒、制药、水电等行业税收监管，抓好房产税、土地使用税、契税、耕地占用税等税种征管，确保税收及时足额入库。严格落实非税收入管控措施，加大对国有股权出让、国有土地有偿使用、国有资产处置的监缴力度，实现非税收入稳定增长。

加强财政预算管理和金融风险防控。认真落实新《预算法》，全面实行绩效管理，不断提高预算执行的及时性、均衡性。进一步优化支出结构，更加注重保运转、保民生、保稳定、促发展，把科学发展绩效奖纳入财政预算，确保重点民生领域支出持续增长。及时清理盘活财政存量资金，深入开展“小金库”专项治理，真正把有限的资金用到刀刃上。以服务供给侧结构性改革为主线，把防风险与强监管结合起来，坚决打击各类违法违规金融活动，切实防范债务危机和金融风险。

强化金融服务保障。全面落实精准扶贫、易地扶贫搬迁和草食畜牧业、设施农业、中药材产业等贴息贷款政策，扩大惠农贷款覆盖面，为群众提供贴心服务。加快金融支撑集中突破，规范“3+1”金融服务体系建设，组建以龙江金融服务公司为核心、九县区融资平台为支撑的国资担保集团，破解中小企业融资担保难题。以服务农村“三变”改革为重点，激活产权交易市场，提升农村“三权”抵押交易平台运营水平。优化金融环境，推动金融机构扩大信贷投放，提升金融支撑保障能力。

进一步提升融资能力。大力推进国有投融资平台建设，加快龙江城乡公司、信通公司等融资平台市场化转型，通过做大实业、股权投资等方式，扩大资产规模，提高运营能力。充分发挥政府投资的引导作用和放大效应，积极争取和用好地方政府债券，大力推广运用“PPP”模式，激发民间投资潜力和创新活力，为全市重大项目建设、特色产业开发和经济社会发展提供资金保障和有力支撑。

九、全面深化改革开放。全面深化改革，激发内生动力是各项事业发展的必然要求。我们要始终把改革创新精神贯穿到各项工作中，把激活内力与借助外力更好地结合起来，切实增强经济社会发展活力。

深化供给侧结构性改革。以提高供给质量和效益为重点，加快推进“三去一降一补”任务落实，推动经济结构调整、转型升级取得新突破。全面落实水泥、煤炭去产能任务，4户煤矿企业要完成技改和机械化改造，加强对疑似“地条钢”企业的监管，严防死灰复燃。把去库存与保障房建设、棚户区改造、新型城镇化结合起来，优化房地产结构，减少房地产库存。认真落实差别化信贷政策，引导企业盘活存量资产，加大股权融资、直接融资力度，降低企业杠杆率。落实一企一策降成本政策，改小扩大、扶弱变强，切实降低实体经济成本和制度性交易成本。坚持问题导向，在脱贫攻坚、基础设施、公共服务设施、生态环境和安全生产等重点领域加大改革力度，着力补齐短板，促进经济社会持续协调健康发展。

深入推进重点领域改革。以简政放权、放管结合、优化服务为突破口，深化机构和事业单位改革，科学合理配置政府部门及内设机构；抓好“接放管服”改革任务落实，优化审批服务，创新监管方式，切实增强政府服务效能。积极推进“互联网+政务服务”，依托陇南发布平台，加大乡村大数据普及应用，建成“只跑一趟、一次办成”的网上政务大厅，使政务服务更亲民、更便民、更惠民。深化商事制度改革，放宽准入限制，完善监管体制，新增市场主体6000户以上。全面完成县区公务用车改革任务。加快农村集体产权制度改革，学习六盘水市农村“三变”改革经验，每个县区确定2个乡镇先行试点，以培育壮大产业为基础，找准改革的路径和办法，走出一条符合陇南实际的农村“三变”改革新路子。深入推进城市综合执法体制改革，完善保障机制，创新治理方式，提升城市管理水平。加快国资国企、医药卫生、供销合作社、国有林场、公路管理等行业改革，进一步激发社会活力。加快推进大众创业、万众创新，积极引进急需紧缺人才，加大本地人才培养力度，构建一批低成本、便利化、全要素、开放式的创业服务平台，让陇南真正成为创业创新者的天堂。

继续扩大开放开发。积极引进和利用外资。加强与“一带一路”沿线国家、城市之间的交流合作，引进国外先进技术，实施一批规模大、效益好、高科技、无污染的好项目。组织市内企业开拓国际市场，设立陇南出入境检验检疫机构，扩大进出口贸易。加强与周边地区的交流合作。以南向通道沿线地区经济联合会、商会为纽带，深化与四川、重庆、贵州、广西、陕西等地区的务实合作，加强与知名企业的战略合作，努力建设区域开放开发共同体。加强文化交流合作。精心筹办乞巧女儿节、白马人民俗文化旅游节、西狭颂旅游节，邀请国内知名专家举办先秦文化、氐羌文化、茶马古道和青泥古道等地方文化论坛，进一步提升陇南的知名度和影响力。

十、全力推进灾后恢复重建。“8.7”暴洪泥石流灾害灾后恢复重建是当前的一项重要政治任务，也是脱贫攻坚和改善民生的重大机遇。我们要统筹推进灾后重建、新型城镇化、美丽乡村、脱贫攻坚工作，使灾区经济社会发展和群众生产生活条件超过灾前水平。

优先抓好农村居民住房重建。按照统一规划设计、群众自建为主的要求，科学编制居民住房恢复重建方案，用足用好易地扶贫搬迁、危房改造、地质灾害避让搬迁、暴洪灾害重建和棚户区改造等各类政策，全面完成4923户居民住房重建任务。重点抓好7个集中安置点和71个重点村恢复重建[29]，启动实施9个乡镇道路、供排水等基础设施建设，把重建村打造成功能完善、居住安全、环境优美、宜游宜业的生态文明新农村。

统筹推进基础设施和公共服务设施恢复重建。加快交通、水利、电力、通信等基础设施重建项目前期工作，启动实施14条国省干线公路、6条县乡道路、344条通村公路和10座便民桥恢复重建，抓好农村安全人饮、河道治理和生态治理等重建项目。全面完成教育、卫生和文化、体育等公共服务设施重建任务，统筹推进旅游、商贸物流和农业、工业等领域恢复重建，切实改善受灾群众生产生活条件。

提升防灾减灾救灾能力。坚持以防为主、防抗救相结合，进一步健全防汛、地震和地质灾害监测预警、应急处置和抢险救灾等防灾减灾救灾体系。完善自然灾害监测预警系统，消除监测盲点。抓好应急避难场所、监测预警和防汛防震基础设施建设，加强地质灾害隐患排查和综合治理[30]，提高城乡基础设施抗灾能力。制定完善应急预案，每年6月8日为防汛抗洪日、5月12日为防震减灾日，集中组织开展科普宣传、应急演练，进一步提升防灾减灾救灾能力。

加强重建项目资金监管。严格执行重建资金监管程序和办法，对涉及土地征占、物资发放、资金分配等工作张榜公示，接受群众监督。认真落实项目法人责任制、招投标制、工程监理制、合同管理制和工程质量终身责任制，加强对灾后重建项目、资金的监督检查和跟踪审计，确保重建资金专款专用，实现阳光重建、廉洁重建。

十一、着力保障和改善民生。增进民生福祉是发展的根本目的。我们要把解决好群众最关心、最直接、最现实的利益问题，作为各项工作的出发点和落脚点，着力夯实民生基础、执政基础，不断满足人民群众日益增长的美好生活需要。

统筹推进教育卫生事业发展。坚持优先发展教育事业，推进城乡义务教育一体化改革，文县、西和县实现义务教育均衡发展；提高教育质量，解决好中小学生课外负担重、城区学校“入学难”和“大班额”等问题，加快发展学前教育，普及高中阶段教育，努力让每个孩子享有公平而有质量的教育。推进城乡医疗卫生一体化改革，整合县乡医疗机构，提高医疗质量和服务水平，解决好“看病难、看病贵”问题，让人民群众享有安全方便价廉的优质医疗服务。加强计划生育服务管理，人口自然增长率控制在9.1‰以内。加快创建国家级医养结合试点市，建成市级医养结合服务中心、8个城市养老机构、10个医养结合示范点和120个老年人日间照料中心[31]，逐步实现老有所养、老有所医。

提高就业和社会保障水平。落实好积极的就业政策，进一步拓宽高校毕业生、城镇困难人员就业渠道，抓好退役士兵安置和权益保障工作，新增城镇就业1.75万人，城镇登记失业率控制在4%以内。落实最低工资标准、最低生活保障标准，实现城乡居民收入持续稳定增长。鼓励社会资金进入养老、医疗等领域。统筹做好社会保险、社会救助、医疗救助、法律援助和特困人员供养等工作，继续推进机关事业单位养老保险制度改革，进一步健全社会保障体系。

大力发展文化体育科技事业。打造一批独具特色、有一定影响力的陇南文化品牌，创作一批精品文艺作品。规划建设陇南大剧院、文化创意产业园，市图书馆、博物馆面向社会免费开放。开工建设陇南市新华书店。建成社区综合文化服务中心 17个，新建“历史再现”工程博物馆11个[32]。创建陇南市国家级卫生城市。开展全民健身活动。加快市体育场建设，建成农村体育场地100个。打造陇南体育品牌，举办有影响的大型体育赛事，积极组织参加省第十四届运动会。培育发展更多的市场科技主体，支持民营科技企业加快发展。瞄准特色产业、科技前沿，大力培育和发展科研院所，加快科技成果转化。

不断提升社会治理水平。深入推进法治陇南建设，扎实开展“七五”普法工作。深化“双查双整”集中行动，健全矛盾纠纷排查化解机制，全面解决信访突出问题。落实粮食安全和菜篮子工程责任制，持续开展食品药品安全专项整治，保障人民群众饮食用药安全。落实价格监管措施，确保市场价格基本稳定。加快民族乡村经济社会发展。推进双拥共建工作。加强老龄、残疾人、档案和公益事业发展，大力支持各民主党派和工、青、妇等群团工作，积极做好留守老人、留守儿童和妇女儿童权益维护工作。深化“平安陇南”建设，依法打击和惩治各类违法犯罪活动，使人民群众的获得感、幸福感、安全感更加充实、更有保障、更可持续。

全力办好民生实事。在全面完成省政府改善民生实事的同时，市政府将集中精力抓好城乡低保指导标准和补助水平、农村特困救助供养补助标准提升，为老年人、农村留守儿童购买人身意外伤害险，为困难弱势群体提供法律援助，在深度贫困村设立5万名公益性岗位等11件实事[33]，让陇南人民生活得更加美好、更加幸福、更加和谐，使美丽陇南建设更加闪耀发光。

各位代表，奋进新时代、开创新征程，对政府工作提出了新的更高要求，我们必须以强烈的责任担当、更加务实的作风，持之以恒加强政府自身建设。一是努力提升服务效能。牢固树立“四个意识”，认真学习贯彻习近平新时代中国特色社会主义思想，在政治立场、政治方向、政治原则、政治道路上始终同以习近平同志为核心的党中央保持高度一致。深入推进“两学一做”学习教育常态化制度化，抓好“不忘初心、牢记使命”主题教育，筑牢信仰之基、把稳思想之魂。自觉践行以人民为中心的发展思想，把关系陇南发展的大事要事落实好，把关系群众冷暖的热点难点问题解决好，切实增强老百姓的获得感、幸福感。进一步解放思想，努力提升谋划发展、推动工作的能力和水平，以实实在在的业绩回报组织的信任和人民群众的期盼，打造组织放心、群众满意的市政府班子。二是全面推进依法行政。坚持把法治精神、法律意识、法制观念贯穿到政府工作的全过程，切实做到依法办事、秉公用权、为民执政。充分发挥市政府决策咨询委员会的作用，把公众参与、专家咨询、风险评估、合法性审查和集体讨论决定作为重大决策的必经程序，进一步提高依法行政水平。自觉接受市人大及其常委会的法律监督、工作监督和市政协的民主监督，广泛听取各方面的意见建议，打造决策民主、依法行政的市政府班子。三是切实改进工作作风。强化政府督查和绩效考核，当好“主攻手”，高效务实、精益求精地做好每一项工作。坚持“严”当头、“效”为要，全面推行责任明确、时限清楚的定事定责限时办理制度，严厉查处不作为、慢作为和乱作为行为，确保政令畅通、令行禁止。大力推行电子政务和网上审批、网上办事，全面深化政务公开，规范政务大厅运行，为群众提供便捷高效服务。不折不扣落实中央八项规定及实施细则精神，弛而不息纠“四风”，紧盯问题抓整改，以良好的作风凝心聚力，打造务实高效、精诚团结的市政府班子。四是不断加强廉政建设。坚决扛起全面从严治党责任，坚持“一岗双责”，严格遵守党的政治纪律、组织纪律、廉洁纪律、群众纪律、工作纪律、生活纪律，全力维护山清水秀的政治生态。发挥好市政府重大行政决策廉政监督委员会的作用，强化行政监察和审计监督，规范土地开发、政府采购、项目资金安排、国有资产转让、重大工程建设招投标管理,严肃查处损害群众利益的不正之风和腐败问题，打造清正廉洁、风清气正的市政府班子。

各位代表，新的目标催人奋进，使命担当引领未来。让我们在以习近平同志为核心的党中央坚强领导下，深入贯彻省委、省政府和市委的各项决策部署，紧紧依靠全市各级组织和广大人民群众，开拓创新、真抓实干，努力开创新时代陇南发展的新局面，为建设幸福美丽新陇南、实现人民群众对美好生活的向往而努力奋斗！
